# Tomato lipocalins mediate ABA (abscisic acid)- and ethylene-dependent regulation of stress tolerance and fruit ripening

**DOI:** 10.5511/plantbiotechnology.25.1023b

**Published:** 2026-03-25

**Authors:** Shoko Kokubo, Miku Tomiyasu, Gang Ma, Chikako Fukazawa, Reiko Motohashi

**Affiliations:** 1 Graduate School of Science and Technology, Shizuoka University, 13-5-1 Johoku, Naka-ku, Hamamatsu, Shizuoka 432-8561,Japan; 2 Applied Life Science Course, Graduate School of Integrated Science and Technology, Shizuoka University, 836 Ohya, Suruga-ku, Shizuoka, Shizuoka 422-8529, Japan; 3 Faculty of Agriculture, Shizuoka University, 836 Ohya, Suruga-ku, Shizuoka, Shizuoka 422-8529, Japan

**Keywords:** abscisic acid, ethylene, lipocalin, phytohormone, tomato

## Abstract

Plant temperature-induced lipocalins (TILs) have been shown to be responsive to heat stress. The expression of TIL in wheat and Arabidopsis is induced by heat shock treatment and cold acclimation, but other responses and functions of lipocalins remain unknown. In this study, we focused on the response of lipocalins to phytohormones in tomato, as *cis*-element analysis revealed the presence of multiple phytohormone-responsive elements involving ethylene, abscisic acid (ABA), and jasmonic acid (JA). The expression levels of *SlTIL1* and *SlTIL2* increased after ABA treatment in young leaves, and tomato plants exhibited enhanced drought stress tolerance 24 h after ABA application. In addition, *SlTIL1* expression increased in tomato fruits at the yellow stage following ABA treatment from the orange stage, thereby accelerating fruit ripening. We compared wild-type plants with overexpressing lines of *SlTIL1*, *SlTIL2*, and *SlCHL* and found that both ethylene gas production and expression of the ethylene synthesis gene *SlACS2* were elevated from the yellow stage in *SlTIL1*-OX and *SlTIL2*-OX lines compared to wild-type. We suggest that ethylene and ABA treatments induce reactive oxygen species (ROS) in tomatoes, to which lipocalins respond by not only contributing to ROS accumulation scavenging but also promoting leaf senescence and fruit ripening.

## Introduction

As tomato fruits mature, the grana lamella structure, an inner membrane structure within the chloroplasts of fruit cells dissolves, while chlorophyll, an antenna protein encapsulated in the thylakoid membrane, is rapidly degraded. These chloroplasts then differentiate into chromoplasts, where lycopene and other carotenoids are synthesized and accumulated, causing the fruit to change color to red (Li and Yuan 2013). “Micro-Tom” (*Solanum lycopersicum* cv. “Micro-Tom”) was used as the experimental tomato in our study, along with “White Beauty”, a white tomato with larger, oval-shaped fruits compared to “Micro-Tom”, and “Black Tomato”, which produces nearly black fruits with shoulders that appear as a mix of green and red. Carotenoid content was measured in each tomato fruit. The results showed that mature fruits of “White Beauty” did not accumulate carotenoids but did accumulate naringenin chalcone, a type of flavonoid. The carotenoid content in mature fruits of “Black Tomato” was about half that of mature “Micro-Tom” fruits. Transmission electron microscopy of plastids in fruit cells from each cultivar revealed chloroplast grana lamella structures in “Micro-Tom” fruits at the Mature Green stage, 30 days after flowering. At the Yellow and Orange stages (32 to 35 days after flowering), the grana lamella structure was degraded, while crystalline and high molecular density membrane structures such as those containing carotenoids were observed. By the Red stage (45 days after flowering), a spiral membrane structure associated with carotenoids was visible.

Small thylakoids similar to those in the Yellow stage of “Micro-Tom” were also observed in mature fruits of “Black Tomato”. In contrast, the mature fruits of “White Beauty” retained plastid structures resembling the Mature Green stage of “Micro-Tom”, indicating a clear difference in plastid development and structure among these tomato cultivars (Suzuki et al. 2015).

To reveal the relationship between plastid differentiation and fruit color and ripening, we conducted a proteomic analysis of plastid proteins in tomato fruits. Plastids were isolated from fruits of different colors using Nycodenz density gradient centrifugation, after which plastid proteins were extracted and separated by two-dimensional electrophoresis. The results indicated that the isoelectric point, molecular mass, and expression levels of temperature-induced lipocalin (TIL) proteins varied. Subsequently, comparison of plastid proteins in fruits of “White Beauty” and “Black Tomato” revealed that levels of the carotenoid-accumulation-related chromoplast-specific carotenoid (CHRC)-associated protein and a plastid lipid-associated protein/fibrillin family protein-like sequence—harpin binding protein 1 were lower than those in “Micro-Tom”. These proteins exhibit properties and functions similar to those of lipocalins, including the ability to transport hydrophobic molecules (Suzuki et al. 2015).

Lipocalins are a protein family widely conserved across animals, plants, and bacteria and are capable of binding small hydrophobic molecules. The crystal structure of lipocalins is highly conserved, featuring a hydrogen-bonded β-barrel composed of eight antiparallel β-strands that enclose the internal ligand-binding site (Dong et al. 2024). Previous genome sequencing results have identified at least 20 distinct types of bacterial lipocalins. One example, the bacterial outer membrane lipoprotein Blc (a lipocalin), is thought to be involved in cellular responses to water stress, such as starvation and high osmotic pressure. Blc differs from other lipocalins in its lack of intramolecular disulfide bonds. It plays a role in membrane biosynthesis and repair and has also been implicated in the propagation of antibiotic resistance genes and the activation of immune responses (Redl and Habeler 2022).

Lipocalins in animals play important roles in immunity and developmental regulation and are involved in responses to various stresses and signaling pathways. In humans, apolipoprotein D (APOD) was the first lipocalin-type glycoprotein identified in 1963 as a distinct component of the human plasma lipoprotein system (Ayrault Jarrier et al. 1963; Do Carmo et al. 2007). APOD expression is induced under several stress conditions, including oxidative stress, inflammatory stress, and UV exposure. It has been shown that APOD expression is suppressed by estrogen, a cell growth promoter, and induced by androgen, a cell growth inhibitor.

In insects, lipocalins such as glial lazarillo offer protection against oxidative stress, as observed in *Drosophila melanogaster* (Sanchez et al. 2006). In plants, lipocalins are classified into TILs and CHLs (Charron et al. 2005). Based on amino acid sequences, structural features, and phylogenetic analyses, TILs and CHLs show homology with evolutionarily related lipocalins, including Blc in bacteria, apolipoprotein D in mammals, and lazarillo in insects. *OsTIL1* in rice maintains cell membrane integrity under oxidative damage during cold stress, making it a promising candidate gene for improving cold tolerance (Ji et al. 2024). In *Arabidopsis*, *AtTIL*1 expression increases under both high and low temperature stress and has been associated with heat stress tolerance in *AtTIL*1 knockout mutants (Chi et al. 2009). AtCHL localizes to the thylakoid lumen of chloroplasts and enhances tolerance to oxidative stress induced by paraquat treatment and drought conditions (Levesque-Tremblay et al. 2009). Based on these findings, we analyzed the function of lipocalins in tomato (Wahyudi et al. 2018, 2020). Two lipocalin genes, *SlTIL1* and *SlTIL2*, were isolated in tomato, and these genes share 84% identity at the amino acid level (Wahyudi et al. 2018). *Cis*-element analysis of the 1,000 bp promoter region upstream of each lipocalin gene revealed elements responsive to abiotic stresses such as temperature and drought. Expression analysis showed that these genes were upregulated under low temperature, salinity, and oxidative stress. In contrast, the expression of *SlTIL2* and *SlCHL* was increased under salinity stress but decreased under high temperature and paraquat stress. *SlCHL* expression also declined during fruit ripening, and *SlCHL* was observed to localize in the chloroplasts (Wahyudi et al. 2018). Subsequently, overexpression (OX) and gene-silenced tomato plants were generated for *SlTIL1*, *SlTIL2*, and *SlCHL*. OX plants displayed early flowering, increased numbers of flowers, inflorescences, and fruits, and exhibited enlarged petioles and fruits compared to wild type. In contrast, gene-silenced plants exhibited delayed flowering and fruit ripening, reduced fruit numbers, and early leaf senescence. The expression levels of superoxide dismutase (SOD) genes, which encode enzymes that degrade ROS, were elevated in OX plants of *SlTILs* and *SlCHLs* compared to the wild type, while they were reduced in gene-silenced lines (Wahyudi et al. 2020).

In tomato fruit, abscisic acid (ABA) synthesis precedes ethylene synthesis (Barry et al. 2000). ABA promotes several ripening processes, including carotenoid accumulation, by regulating transcription factors involved in ethylene biosynthesis and signaling (Miyakawa and Tanokura 2011; Mou et al. 2016). For example, ABA has been reported to directly participate in cell wall catabolism, thereby promoting fruit softening through the regulation of related enzymes and gene expression (Sun et al. 2012). Furthermore, it has been reported that exogenous ABA treatment may promote tomato fruit maturation by enhancing carotenoid biosynthesis and chlorophyll degradation (Barickman et al. 2014).

In another study, exogenous ABA treatment of tomato fruit promoted fruit ripening and ethylene emission, while treatment with nordihydroguaiaretic acid (NDGA) inhibited both processes (Mou et al. 2016). Investigation of tomato fruit treated with 1-MCP (an ethylene inhibitor) immediately after ABA exposure revealed that ethylene may be essential for the induction of ABA biosynthesis and signaling at the onset of fruit ripening (Mou et al. 2016). Additionally, several transcription factors (TFs), such as *MADS-RIN*, *TAGL1*, *CNR*, and *NOR*, known regulators of ethylene synthesis and sensitivity, were also found to be ABA-responsive, suggesting that ABA may influence ethylene action through regulation of these TFs (Cheng et al. 2009; Ito et al. 2017; Mou et al. 2016). Therefore, understanding the interplay between ABA and ethylene actions may provide deeper insight into carotenoid biosynthesis and fruit maturation during tomato ripening. Recent studies have shown that phytoene overproduction initially interferes with photosynthesis, acting as a metabolic threshold switch that weakens chloroplast identity (Llorente et al. 2020). Phytoene also promotes carotenoid synthesis and contributes to fruit maturation. Since a burst of ROS has been shown to trigger chromoplast differentiation (Morelli et al. 2023), tomato lipocalins may also play a role in chromoplast differentiation. However, the relationship between lipocalins and carotenoid pigments remains to be elucidated. In this study, we treated “Micro-Tom” tomato plants with exogenous phytohormones, ethylene, jasmonic acid (JA), and ABA to investigate the relationship between these hormones and tomato lipocalins, which are the focus of this study, as well as to observe the corresponding physiological responses in tomato plants.

## Materials and methods

### Plant materials

“Micro-Tom” (*S. lycopersicum* cv.) was used in this study. The plants were grown in an incubation room under standard culture conditions (60–80% relative humidity, 24±2°C, 16/8 h light/dark photoperiod, and 150 µmol m^−2^ s^−1^ light intensity). The following samples were collected from tomato plants: leaves from 5–6-week-old plants. Based on days post-anthesis (DPA), the ripening process of “Micro-Tom” fruit was divided into four stages: Mature Green stage (30–33 DPA); Yellow stage (breaker stage) (32–35 DPA); Orange stage (35–40 DPA); and Red stage (41–45 DPA) (Suzuki et al. 2015). Leaves and fruits at different ripening stages were collected, immediately frozen in liquid nitrogen, and stored at −80°C until use.

### The analysis of the upstream genomic region of lipocalins in tomato

To predict regulatory and stress-responsive elements in the upstream regions 1,800 bp of *SlTILs* and *SlCHL*, the online tools PlantCARE (https://bioinformatics.psb.ugent.be/webtools/plantcare/html/) and Expression Atlas (https://www.ebi.ac.uk/gxa/home (Accessed Sep 16, 2024)) were used to identify the cis-acting elements (Lescot et al. 2002; Mall et al. 2023). The putative transcription start sites were identified using the Neural Network Promoter Prediction tool (https://www.fruitfly.org (Accessed Sep 16, 2024)), and a putative TATA box was found in the sequence.

### Ethylene response of lipocalins in tomato

“Micro-Tom” wild-type (WT) plants were grown for five weeks after sowing, as previously described. Ethylene treatments were performed in a sealed 48 l acrylic chamber (dimensions: L50 cm×W30 cm×H32 cm). WT plants were treated with either 50 or 100 ppm ethylene gas using a medical syringe, administered three times. Plants were then incubated at 27°C and 49% relative humidity. Ethylene-treated leaves were sampled at 0, 2, and 6 h post-treatment, immediately frozen in liquid nitrogen, and stored at −80°C until use.

### JA response of lipocalins in tomato

To evaluate the effect of exogenous methyl jasmonate (MeJA) treatment, young leaves of WT plants were treated five weeks after sowing. MeJA treatment was performed by dissolving 38.6 µM MeJA in 0.01% ethanol. Distilled water with 0.01% ethanol was used as a control. Leaf samples were collected in triplicate at 1, 2, 3, 5, and 7 h after treatment, frozen in liquid nitrogen, and stored at −80°C until use.

### ABA response of lipocalins in tomato

To assess the effect of exogenous ABA treatment, young leaves were treated five weeks after sowing, and fruits were treated at the mature green stage of WT plants. ABA treatments were carried out by dissolving 50 or 100 µM ABA in 1% methanol. A 1% methanol solution was used as the control. Leaf samples were collected in triplicate at 0, 2, 4, 6, 8, and 24 h after treatment, then immediately frozen in liquid nitrogen and stored at −80°C.

Fruits from plants grown at 24°C and 50–80% RH were treated with 0.5 ml of 50 or 100 µM ABA dissolved in 1% methanol. Twenty tomato fruits at the Mature Green (MG) stage were treated with each concentration. These fruits were then harvested at the Yellow, Orange, and Red stages and immediately frozen in liquid nitrogen as described above.

Thermal imaging was used to investigate whether ABA treatment increased the surface temperature of leaves and fruits. Images were captured every 10 min for 24 h using a Testo 885 thermal camera (Testo SE & Co. KGaA, Japan), with a temperature range of −30 to 100°C and thermal sensitivity of <0.10°C. Images were taken from a distance of approximately 15 cm, using an emissivity value (ε) of 0.97. Average surface temperatures of leaves and fruits were analyzed using Testo IRSoft V4.1 software, with each image capturing all genotypes and treatments simultaneously.

### Fruit ripening and expression of ethylene biosynthesis genes

Ethylene is a key plant hormone that regulates the ripening of climacteric fruits, and several transcription factors serving as critical regulators of fruit ripening have been identified in tomato, a model system for such fruits. In this study, the expression of four ethylene biosynthesis genes (i.e., *SlACO1*, *SlACS1*, *SlACS4*, and *SlACS2*) was analyzed using the RT-PCR method (Supplementary Table S1) and compared across OX lines of *SlTIL1*, *SlTIL2*, *SlCHL*, and WT samples of young leaves or fruits at various ripening stages (Wahyudi et al. 2020).

### Fruit ripening and expression of carotenoid biosynthesis genes

Carotenoids, a class of terpenoids composed of 40 carbon skeletons, are synthesized via the MEP pathway (Liu et al. 2015). During tomato fruit development and ripening, carotenoid biosynthesis is regulated by phytoene desaturase (*SlPDS*) and phytoene synthase (*SlPSY1*) (Enfissi et al. 2005; Lois et al. 2000). In this study, to explore the relationship during *SlTIL1*, *SlTIL2* and carotenoid synthase genes, the expression of nine carotenoid-related genes (i.e., *PSY1*, *PDS*, *ZDS*, *CRTISO*, *CYC-B*, *VED*, *ZEP*, *NCED1*, *NCED2*, *Actin* (Solyc10g080500) and 18SrRNA (NCBI Accession No. OK073663.1)) (Yuan et al. 2022; Zhang et al. 2009) was assessed via RT-PCR (Supplementary Table S1) and compared among OX lines of *SlTIL1*, *SlTIL2*, *SlCHL*, and WT fruits collected at each ripening stage (Wahyudi et al. 2020).

### RNA extraction and qRT-PCR analysis

Total RNA from all tissues was extracted using the RNeasy Plant Mini Kit (QIAGEN Co., Ltd., Germany), following the manufacturer’s protocol. This RNA was then used for reverse transcription, according to the instruction manual of the PrimeScript™ High Fidelity RT-PCR Kit (#R0022A, TaKaRa Co., Ltd., Japan). The qRT-PCR of *SlTIL1*, *SlTIL2*, *SlPDS*, and *SlPSY1* transcripts was carried out using TB Green® Premix Ex Taq™ II (#RR820A; TaKaRa) in a 25 µl reaction volume, consisting of 12.5 µl TB Green mix, 0.5 µl of 20 µM of each primer (Supplementary Table S1), 9 µl of distilled water, and 2.5 µl of cDNA (from total RNA at 1,000 ng µl^−1^). The qRT-PCR was performed on a TaKaRa Thermal Cycler Dice® Real Time System III. The tomato housekeeping gene *SlActin* or *18SrRNA* and the comparative 2^−ΔΔCt^ method (Pfaffl 2001) were used for normalization and relative expression analysis, respectively.

### Ethylene measurements

Ethylene production by whole fruit was measured by placing five fruits into 0.1545 l airtight glass bottles for 1 h at 25°C. After incubation, 1 ml of headspace gas was withdrawn and injected into a gas chromatograph (GC-2014; SHIMADZU Co., Ltd., Japan) equipped with a flame ionization detector. Ethylene production was expressed in nl g^−1^ FW h^−1^. The column and detector temperatures were set to 100°C and 200°C, respectively; the nitrogen carrier gas flow rate was 50 ml min^−1^, and the hydrogen pressure was 0.6 kPa (Ma et al. 2021).

## Results

### *Cis*-element analysis of the upstream genomic region of *SlTILs* and *SlCHL*

In silico analysis of the genomic sequences upstream of the start codon (ATG) of *SlTILs* and *SlCHL* was conducted of using PlantCARE and BLAST® NCBI online tools to predict various *cis*-acting elements (Supplementary Figure S1). A total of 35 *cis*-acting elements were identified within the promoters of *SlTILs* and *SlCHL*, and these were classified into four categories: hormone response, light response, plant development, and stress response (Supplementary Figure S1). The results showed that the promoters of *SlTILs* and *SlCHL* contained a greater number of light response and stress response elements, such as Box 4, G-box, heat shock elements (HSEs), MYB, and MYC elements (Supplementary Figure S1). Additionally, the promoters also included several elements related to plant development and hormone response, including the AAGAA-motif, ABREs, ERE, and MeJA response elements, suggesting the functional diversity of tomato *SlTILs* and *SlCHL* genes.

### Response of lipocalins to ethylene treatment

**Figure 1:**
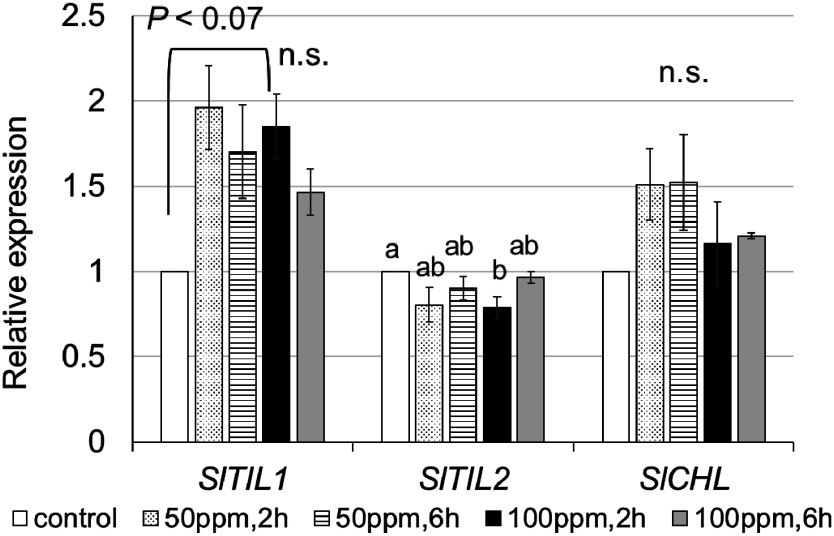
Figure 1. Expression of lipocalin genes in wild-type (WT) leaves treated with ethylene. Relative expression values represent the mean±S.D. of three biological replicates. The relative expression level of control was set to 1. Different letters indicated values significantly different ANOVA followed by Tukey’s multiple comparisons test (*p*<0.05); n.s., not significant (*p*>0.05).

**Figure 2:**
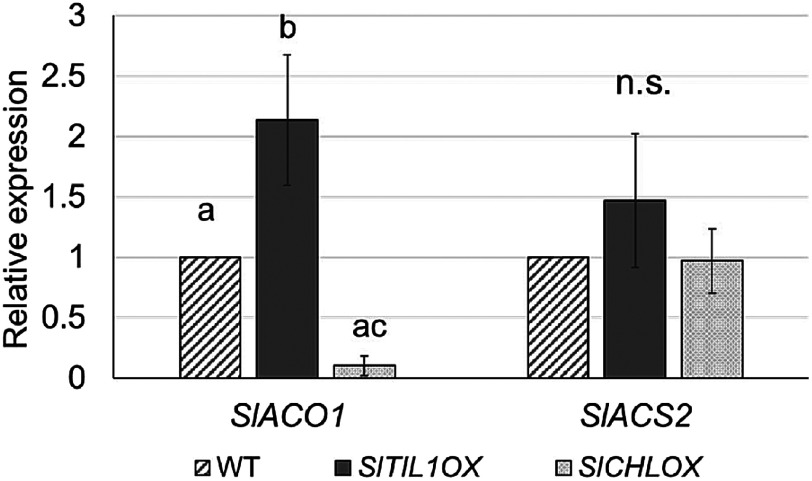
Figure 2. Expression patterns of two ethylene biosynthesis genes, *SlACO1* and *SlACS2*, in *SlTIL1*OX and *SlCHL*OX plants. Relative expression values represent the mean±SD of three biological replicates. The relative *SlACO1* or *SlACS2* expression level in WT was set to 1. Different letters indicate statistically significant differences (*p*<0.05) based on Tukey’s multiple comparisons test; n.s., not significant (*p*>0.05).

*Cis*-elements associated with ethylene response were identified in the promoter regions of all lipocalin genes; therefore, ethylene treatment experiments were conducted on WT young leaves. As a result, the mRNA levels of *SlTIL1* and *SlCHL* increased in leaves treated with 50 or 100 ppm ethylene for 2 h compared to the untreated control (Figure 1). In contrast, *SlTIL2* mRNA did not accumulate in ethylene-treated leaves. Based on the results in Figure 1, we further examined whether *SlTIL1* and *SlCHLOX* plants responded to ethylene by evaluating the expression of *SlACO1* and *SlACS2*, key genes in ethylene biosynthesis. We observed a marked accumulation of *SlACO1* in *SlTIL1*OX plants (Figure 2), suggesting that *SlTIL1* may be involved not only in the ethylene response but also in ethylene biosynthesis.

### Response of lipocalins to JA treatment

In recent years, research on plant abiotic stress has increasingly focused on the JA metabolic pathway. JA plays a crucial role in plant responses to various environmental stresses, including mechanical injury, disease, insect attack, drought, salinity, and extreme temperatures (Shang et al. 2024). In this study, *SlTIL1* expression tended to decrease at 2 h after MeJA treatment, while *SlCHL* expression significantly declined at 5 h (Supplementary Figure S2). Subsequently, *SlTIL1* expression increased at 7 h. In contrast, *SlTIL2* expression was not affected by MeJA treatment. However, the expression of all lipocalin genes tended to recover by 7 h post-treatment.

### Response of lipocalins to ABA treatment

**Figure 3:**
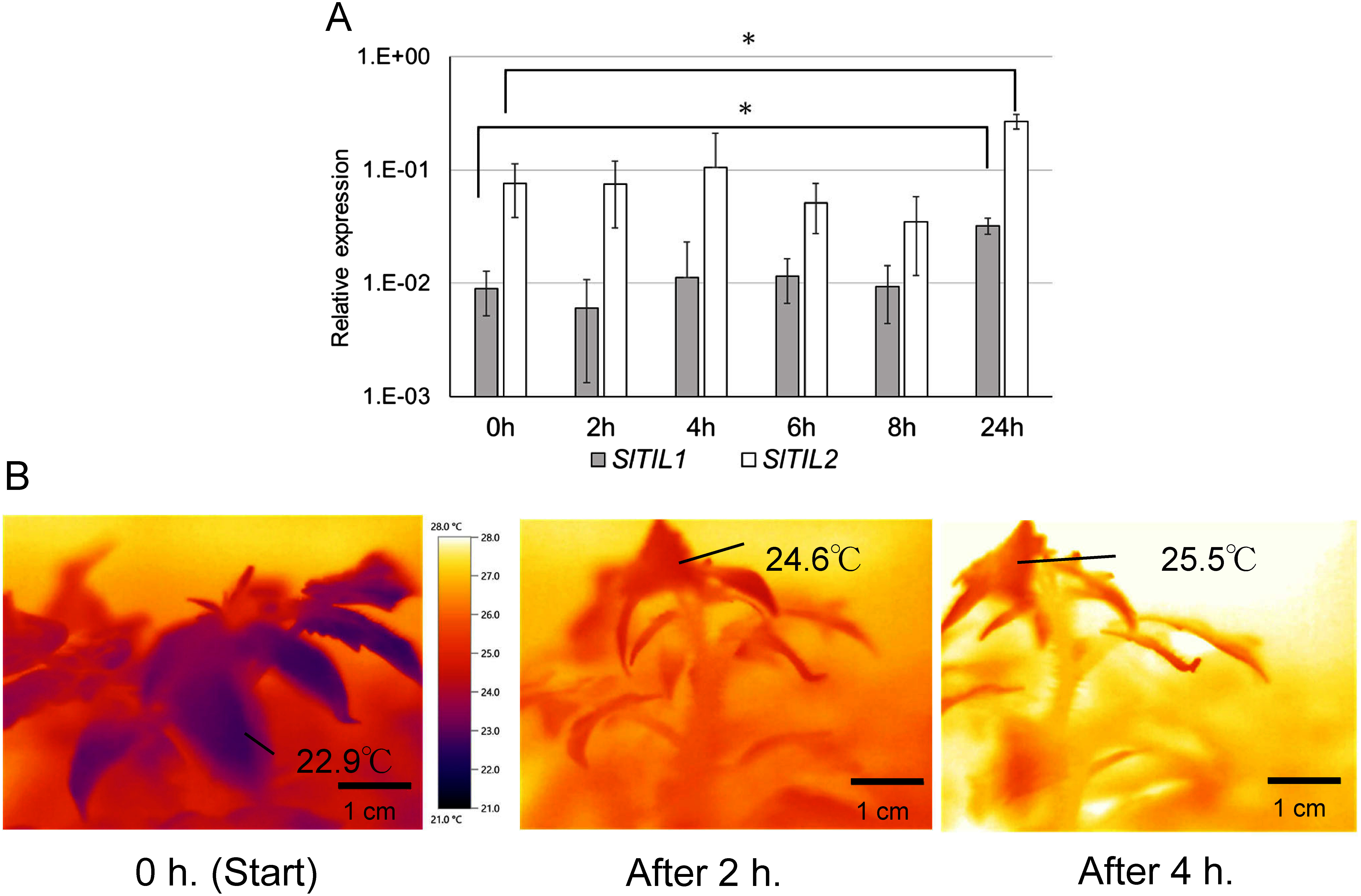
Figure 3. Response of lipocalins to ABA treatment in leaves. (A) Expression of *SlTIl1* and *SlTIl2* in WT young leaves treated with 50 µM ABA. Relative expression values represent the mean±SD of three biological replicates. Asterisks indicate statistically significant differences compared to 0 as determined by ANOVA and a two-tailed Student’s *t*-test (*p*<0.05). (B) Thermal image of WT young leaves treated with 50 µM ABA. The color scale indicates leaf surface temperature. The scale bar represents 1 cm.

We focused on *SlTIL1* and *SlTIL2*, which in our previous study showed changes in expression under abiotic stress conditions (Wahyudi et al. 2020). Their expression was examined in leaves from 5-week-old seedlings treated with 50 and 100 mM ABA (Figure 3A, Supplementary Figure S3). The expression of *SlTIL1* and *SlTIL2* tended to increase between 0 and 4 h, followed by a decline between 4 and 8 h. After 50 µM ABA treatment, both genes showed significantly increased expression levels (Figure 3A). In thermal images, the surface temperature of the leaf was 22.9°C immediately after ABA application and rose to 25.5°C after 4 h (Figure 3B, Supplementary Figure S4). ABA-induced stomatal closure led to an elevation in leaf temperature, accompanied by an upward movement of leaves, a phenomenon known as hyponastic growth.

**Figure 4:**
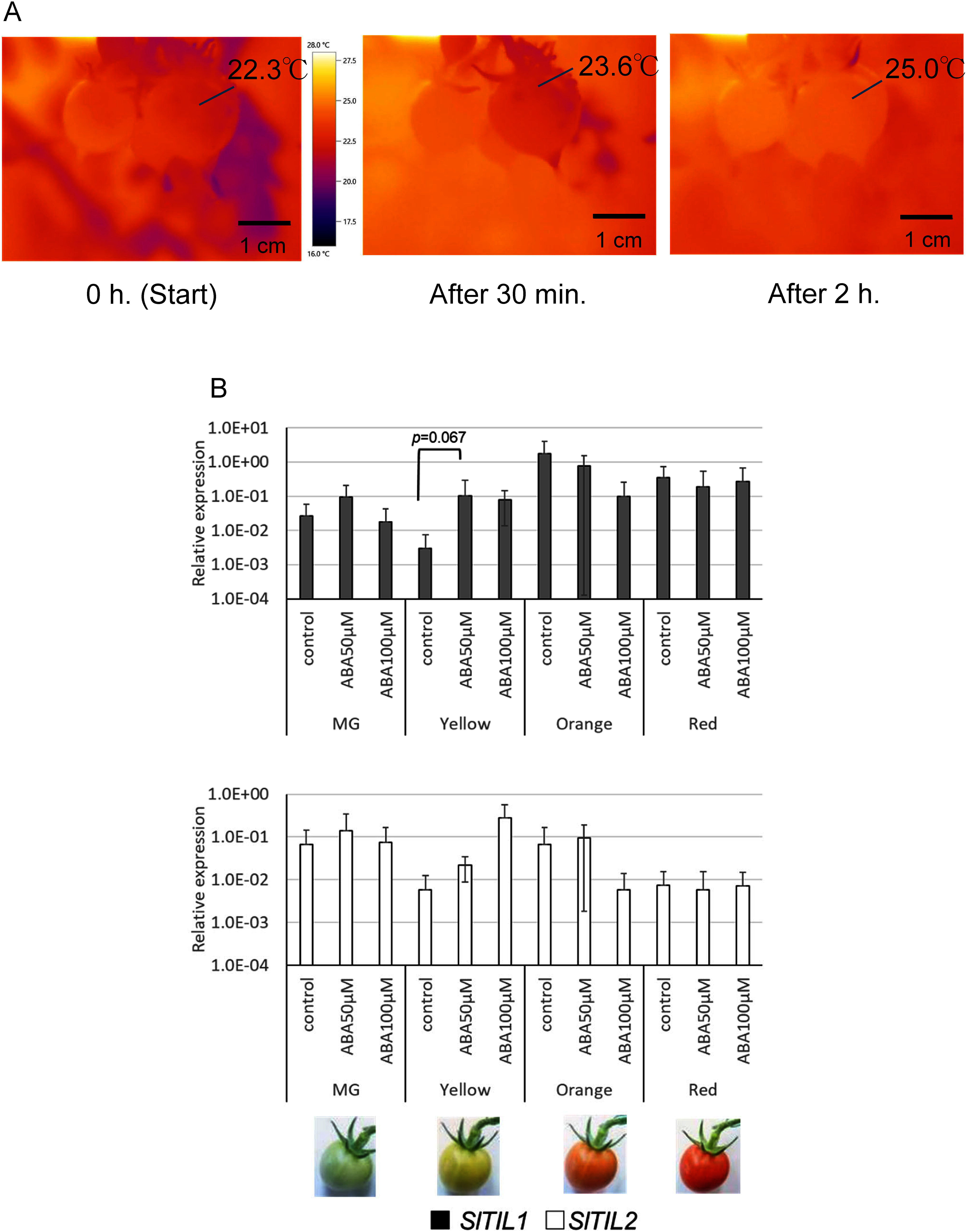
Figure 4. Response of lipocalins to ABA treatment in fruits. (A) Thermal image of mature green (MG) fruits treated with 50 µM ABA. The color scale indicates fruit surface temperature. The scale bar represents 1 cm. (B) Expression of *SlTIl1* and *SlTIL2* in MG fruits treated with 50 µM ABA across different ripening stages: MG (mature green), Yellow, Orange, and Red. Expression levels were analyzed by qRT-PCR (Supplementary Table S1) and are shown relative to control samples. Data represent the mean±SD of *n*=3 fruits. Italicized numbers indicate *p*-values from two-tailed Student’s *t*-test comparing treated and control samples.

When fruits were treated with 50 µM exogenous ABA, their surface temperatures were monitored for 24 h using the same thermal camera described above. The surface temperature was 22.3°C immediately after ABA treatment and increased to 25°C after 2 h (Figure 4A). Fruit ripening began 7 days after treatment, though the bottoms of the fruits remained green due to the direction of spraying from the shoulders. By 9 days after treatment, most fruits had ripened. Softening of the fruits was observed beginning at the Yellow stage; however, the pericarp became firmer. The expression of *SlTIL1* significantly increased in ABA-treated fruits from the Yellow stage onward and was associated with accelerated fruit ripening (Figure 4B).

**Figure 5:**
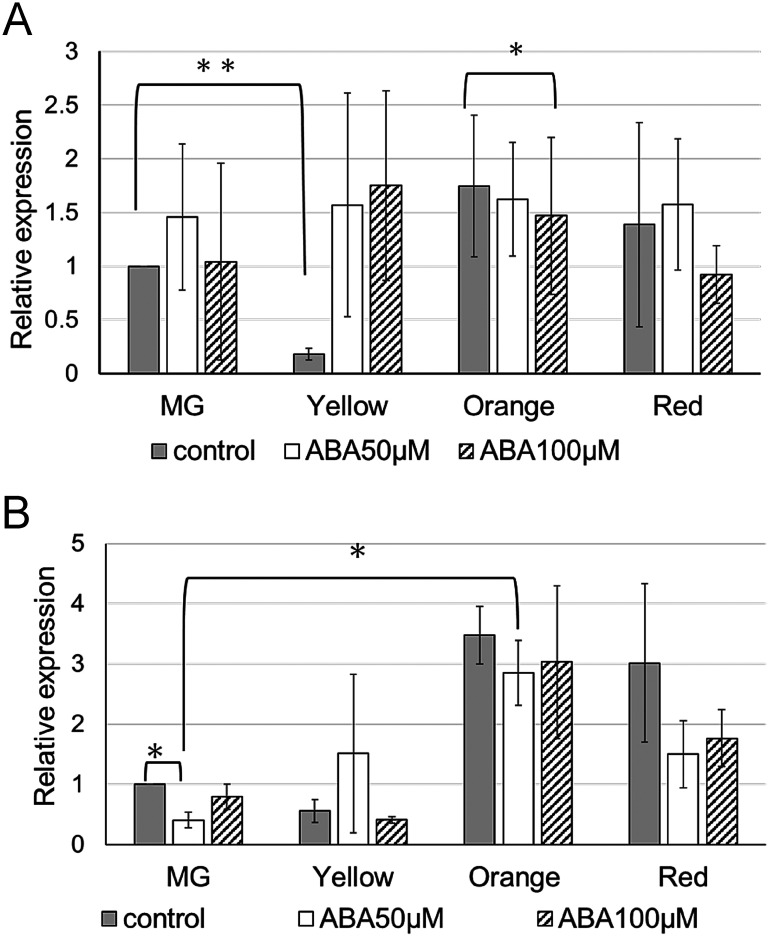
Figure 5. Expression of ethylene biosynthesis genes in WT tomato fruits treated with 50 µM ABA. (A) *SlACO1*, (B) *SlACS2*. Relative expression values represent the mean±SD of three biological replicates. The relative MG stage expression level in WT was set to 1. Asterisks indicate statistically significant differences compared to control fruits (* *p*<0.05, ** *p*<0.01) based on two-tailed Student’s *t*-test.

Ethylene plays a key role in fruit ripening, and the synthesis of S-adenosylmethionine (SAM) from methionine is a conserved reaction across organisms. When 1-aminocyclopropane-1-carboxylic acid (ACC) synthases (ACSs) are expressed, ACC is rapidly produced from SAM, and ethylene is subsequently generated by ACC oxidase (ACO) (Barry et al. 2000; Tatsuki 2007). As a result, the expression of *SlACS2* couldn’t be reliably detected in this study (Tatsuki 2007). However, *SlACO1*, the final gene in the ethylene biosynthesis pathway, was more highly expressed in ABA-treated fruits at the Yellow stage compared to the control, suggesting that ethylene biosynthesis was enhanced by ABA treatment (Figure 5A, B). Furthermore, ripening occurred 3 days earlier in fruits treated with 50 µM ABA than in control fruits (Supplementary Figure S5). Thus, the expression of ethylene biosynthesis genes *SlACO1* and *SlACS2* was induced by ABA treatment during the Yellow stage (Figure 5A, B).

### Expression of ethylene biogenesis genes and ethylene production

**Figure 6:**
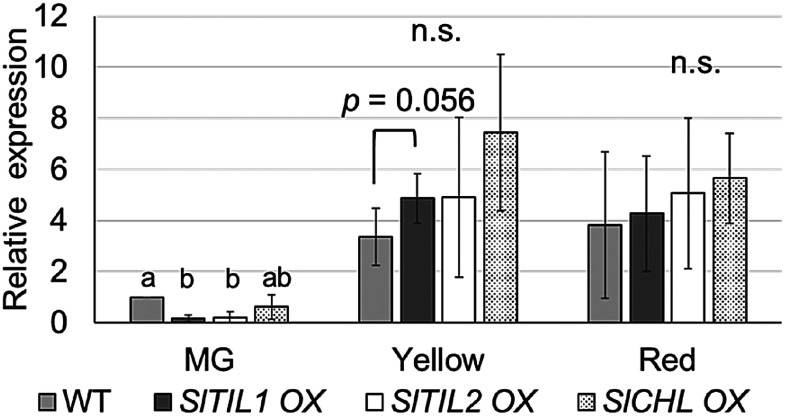
Figure 6. Fruit ripening and expression of the ethylene synthase gene *SlACS2* in *SlTIL1*OX, *SlTIL2*OX, and *SlCHL*OX lines compared with wild-type (WT) plants. Each value represents the mean±SD of three biological replicates. The relative MG stage expression level in WT was set to 1. Different letters indicate statistically significant differences (*p*<0.05) based on Tukey’s multiple comparisons test; n.s., not significant (*p*>0.05).

To investigate the relationship between fruit ripening and the expression of ethylene synthesis genes, *SlACS1*, *SlACS2*, *SlACO1* and *SlACS4* (Mou et al. 2016), were analyzed at three key fruit maturation stages MG, Yellow, and Red using WT and overexpressed *SlTIL1*, *SlTIL2*, and *SlCHL* plants. Since no significant difference in lipocalin gene expression was observed between the Orange and Red stages following ABA treatment (Figure 5), the analysis was focused on the MG, Yellow, and Red stages. The results showed that *SlACS2* expression changed significantly from the Yellow to Red stages. In particular, *SlACS2* expression in *SlTIL1*OX plants was higher at the Yellow stage than in WT. Similarly, in other lipocalin OX lines, *SlACS2* expression was also elevated at the Yellow stage compared to WT (Figure 6, Supplementary Figure S6).

**Figure 7:**
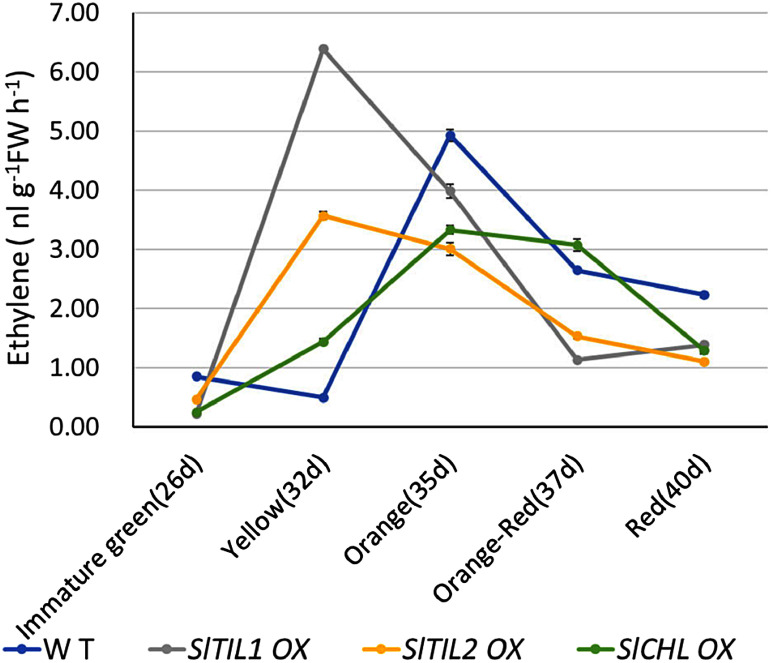
Figure 7. Changes in ethylene production during fruit ripening in tomato flesh. Fruit samples were collected from *SlTIL1*OX, *SlTIL2*OX, *SlCHL*OX, and WT lines at five developmental stages: Immature green (26 DPA), Yellow (32 DPA), Orange (35 DPA), Orange to Red (37 DPA), and Red (39 DPA). Each value represents the mean±SD of three biological replicates.

Fruits from WT and lipocalin overexpressing lines (*SlTIL1*OX, *SlTIL2*OX, and *SlCHL*OX) were collected at different ripening stages to measure ethylene production. The results showed that, consistent with the expression patterns of ethylene biosynthesis genes described above (Figure 6, Supplementary Figure S6), ethylene production was higher at the Yellow stage in *SlTIL1*OX and *SlTIL2*OX fruits compared to WT (Figure 7). In contrast, ethylene production in WT peaked at the Orange stage. As the lipocalin OX plants were previously reported to exhibit earlier flowering (Wahyudi et al. 2018), the increase in ethylene production also occurred earlier in these plants than in WT.

### Fruit ripening and expression of carotenoid synthase genes

**Figure 8:**
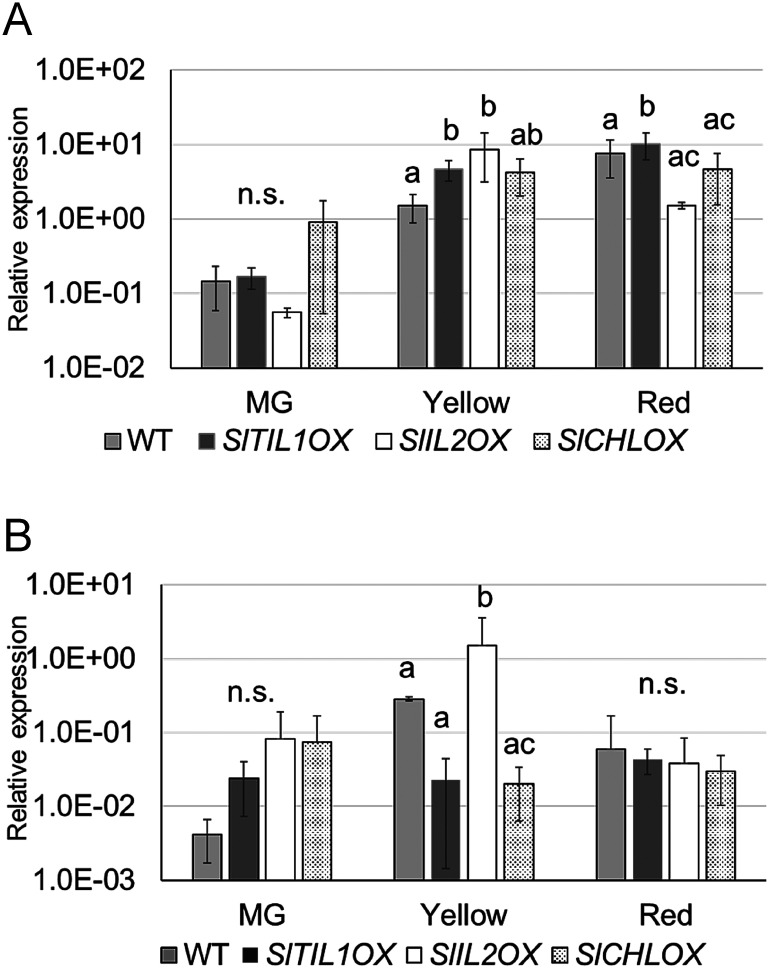
Figure 8. Fruit ripening and expression of carotenoid biosynthesis genes in *SlTIL1*OX, *SlTIL2*OX, and *SlCHL*OX lines compared with wild-type (WT) plants. (A) *SlPSY1*, (B) *SlPDS*. Expression levels were analyzed by qRT-PCR. Each value represents the mean±SD of three biological replicates. Different letters indicate statistically significant differences (*p*<0.05) based on Tukey’s multiple comparisons test; n.s., not significant (*p*>0.05).

The overexpression lines of *SlTIL1*, *SlTIL2*, and *SlCHL* were used to examine nine major genes involved in the carotenoid biosynthesis pathway—*SlPDS*, *SlPSY1*, *SlNCED1*, *SlNCED2*, *SlCRTISO*, *SlCycb-2*, *SlVDE*, *SlZDS*, and *SlZEP* (Supplementary Table S1, Figure 8, Supplementary Figure S7). Among these, *SlPDS* and *SlPSY1*, which encode rate-limiting enzymes for carotenoid synthesis, were further analyzed using real-time PCR due to significant differences observed in RT-PCR. The results revealed that *SlPSY1* expression was significantly higher in *SlTIL1*OX and *SlTIL2*OX plants than in WT at the Yellow stage (Figure 8A), and expression in other overexpression lines also tended to be higher than in WT. Additionally, *SlPDS* expression was significantly increased in *SlTIL2*OX plants at the Yellow stage compared to WT (Figure 8B). These results suggest that carotenoid synthesis may occur earlier in lipocalin OX plants than in WT, possibly due to their earlier flowering time (Wahyudi et al. 2018; Yazdani et al. 2019).

## Discussion

The lipocalin protein family, part of the calycin superfamily, comprises extracellular transport proteins that have recently been explored for their functional diversity (Flower 1996). Our *cis*-element analysis of the promoter regions of the lipocalin genes revealed the presence of numerous elements related to light response, HSEs, ethylene response, ABA response, and various stress responses (Supplementary Figure S1). Specifically, MYC (stress response) and G-box (light response) elements were detected in the promoter regions of *SlTIL1* and *SlCHL*, while MYB elements related to stress response were more frequently found in the *SlTIL2* promoter compared to *SlTIL1* and *SlCHL* (Supplementary Figure S1). In addition, stress response-type *cis*-acting elements, such as MYB and MYC, along with the G-box (light response) and ABRE (ABA response), were identified in *AtTIL1*, with the MYB element being more abundant in *AtTIL1*. In apples (*Malus domestica*), which are also climacteric fruits, the *APOD* gene contained MYB and MYC elements in nearly equal proportions (Supplementary Figure S1). These differences in *cis*-acting elements among *TILs* may be attributed to variations in the timing, tissue specificity, and type of stress response associated with each lipocalin gene. Furthermore, *SlTIL1* contains only one HSE, while *SlCHL* and *SlTIL2* possess several HSEs within the promoter region between −50 and −1,000 bp. HSEs are believed to enhance gene expression not only under heat stress but also in response to various other abiotic stresses, through interaction with specific stress-responsive TFs (Sato et al. 2024; Maruyama et al. 2016). Our previous study indicated that increased expression of *SlTILs* and *SlCHL* was effective in reducing ROS accumulation under heat stress conditions (Wahyudi et al. 2020), suggesting that the rapid response to heat stress may be mediated by the presence of HSEs in their promoter regions (Supplementary Figure S1). Therefore, HSEs may also play a role in mediating the phytohormone response and abiotic stress regulation of lipocalins, and further studies are warranted to deepen our understanding.

In this study, we investigated the expression of lipocalin genes in response to ABA, ethylene, and JA treatments to analyze the relationship between phytohormones and lipocalins. The expression of *SlTILs* was not clearly upregulated following MeJA treatment of tomato leaves (Supplementary Figure S2). This suggests that *SlTILs* are not strongly induced by MeJA, even though JA-responsive *cis*-acting elements are conserved in the promoter regions of *SlTIL1* and *SlTIL2*. Ding et al. (2022) demonstrated that JA treatment under cold stress conditions increases the expression of *SlNCED2*, an ABA biosynthesis gene, thereby promoting ABA accumulation and enhancing cold tolerance. This JA-induced ABA biosynthesis is facilitated by MYC2 binding to the promoter region of *SlNCED2*. Specifically, two G-box elements were identified within 2 kbp upstream of the *SlNCED2* promoter, and MYC2 binding to these G-box sites was shown to activate *SlNCED2* expression and ABA production in response to cold stress (Ding et al. 2022). Reporter region analyses of *AtTIL1* and *APOD* in apple also revealed the presence of G-box (core sequence: CACGTG) motifs, which are recognized by MYC2, and similar G-box elements are found in the promoter regions of *SlTIL1* and *SlCHL* in tomato. This suggests a potential role for G-box–MYC2 interactions in linking JA signaling with lipocalin gene expression. Therefore, further experiments are needed to determine whether JA can initiate interactions with stress-responsive TFs such as MYC2, leading to ABA accumulation and subsequent induction of lipocalin expression in tomato.

In contrast, *SlTIL1* mRNA accumulated in young leaves as early as 2 h after ethylene treatment (Figure 1), and both *SlTIL1* and *SlTIL2* were highly expressed in leaves 24 h after ABA treatment (Figure 3A). These results indicate that *SlTILs* are involved in stress defense mechanisms in leaves, particularly in response to ABA and ethylene. Our findings suggest that ABA treatment induced stomatal closure, leading to increased intercellular temperature and upward leaf movement—a process known as hyponasty, which contributes to thermomorphogenesis (Figure 3B, Supplementary Figure S5) (Quint et al. 2016). This rise in temperature may have also triggered the expression of *SlTILs*. However, ethylene treatment promoted senescence with ROS resulting from excess light exposure and decreased photosynthetic capacity. In response, the expression of *SlTIL1* and *SlCHL* increased to help scavenge the ROS. In *SlTIL1*OX and *SlCHL*OX plants, the expression of *SlSODs* (*S. lycopersicum* superoxide dismutases) was elevated in the leaves, effectively reducing ROS accumulation (Wahyudi et al. 2020). Furthermore, structural comparisons of lipocalins were conducted using TMHMM (https://services.healthtech.dtu.dk/services/TMHMM-2.0/ (Accessed Jul 3, 2025)), a transmembrane domain prediction tool. The results showed that SlTIL1 and SlTIL2 each possess one transmembrane domain, while SlCHL lacks this domain (Supplementary Figure S8A, B, C). Based on predicted transmembrane helix structures, the model for SlTIL2 was more reliable than that of SlTIL1, and both proteins shared a hydrophobic proline-rich (HPR) motif located within the transmembrane domain (Mall et al. 2023). SlTIL1 and SlTIL2 also share 84% amino acid sequence identity (Wahyudi et al. 2018). These differences may contribute to variation in the formation of hydrophobic cavities composed of HPR motifs and eight β-sheets, which can affect membrane biosynthesis and repair, transport of hydrophobic small molecules, and stress responses. Notably, SlTIL*2* exhibited higher expression than *SlTIL1* under ABA treatment (Figure 3A).

In the present study, the expression of *SlTIL1* generally increased during fruit maturation from the Orange to the Red stage in WT plants (Figure 4B). In fruits treated with ABA, *SlTIL1* expression was notably accelerated at the Yellow stage (Figure 4B). At this same stage, the expression of the ethylene biosynthesis genes *SlACO1* and *SlACS2* also increased prior to the onset of ripening (Figure 5A, B). These expression patterns suggest that exogenous ABA treatment promotes fruit ripening, as well as the expression of ethylene biosynthesis genes. A previous study demonstrated that ABA could enhance ethylene production and signaling by regulating key genes such as *SlACS4*, *SlACO1*, and *SlETR6* (Mou et al. 2016), findings that are generally consistent with our results (Figure 5A, B, Supplementary Figure S6). In addition, the results of this study suggested that overexpression lines of lipocalins (*SlTIL1OX*, *SlTIL2OX*, and *SlCHLOX*) might enhance *SlACS2* expression compared to WT plants, thereby contributing to ethylene biosynthesis (Figures 6, 7). On the other hand, recent research has revealed that fruit ethylene production involves not only *SlACS2* but also a significant contribution from *SlACS4* (Li et al. 2025). Therefore, further research will be necessary to deepen our understanding of the molecular mechanisms involved in ethylene signaling pathways. Moreover, elevated temperature contributes to ethylene production and promotes ROS accumulation. In banana fruit, *MaEIL9*, a component of ethylene signaling stimulated by ethylene itself, activates the expression of carotenogenic genes and the transcriptional regulator MaSPL16 by binding to their promoters. This regulation contributes to carotenoid biosynthesis in both banana and tomato, supported by stable and transient transformation technologies (Bharath et al. 2021; Zhu et al. 2023).

**Figure 9:**
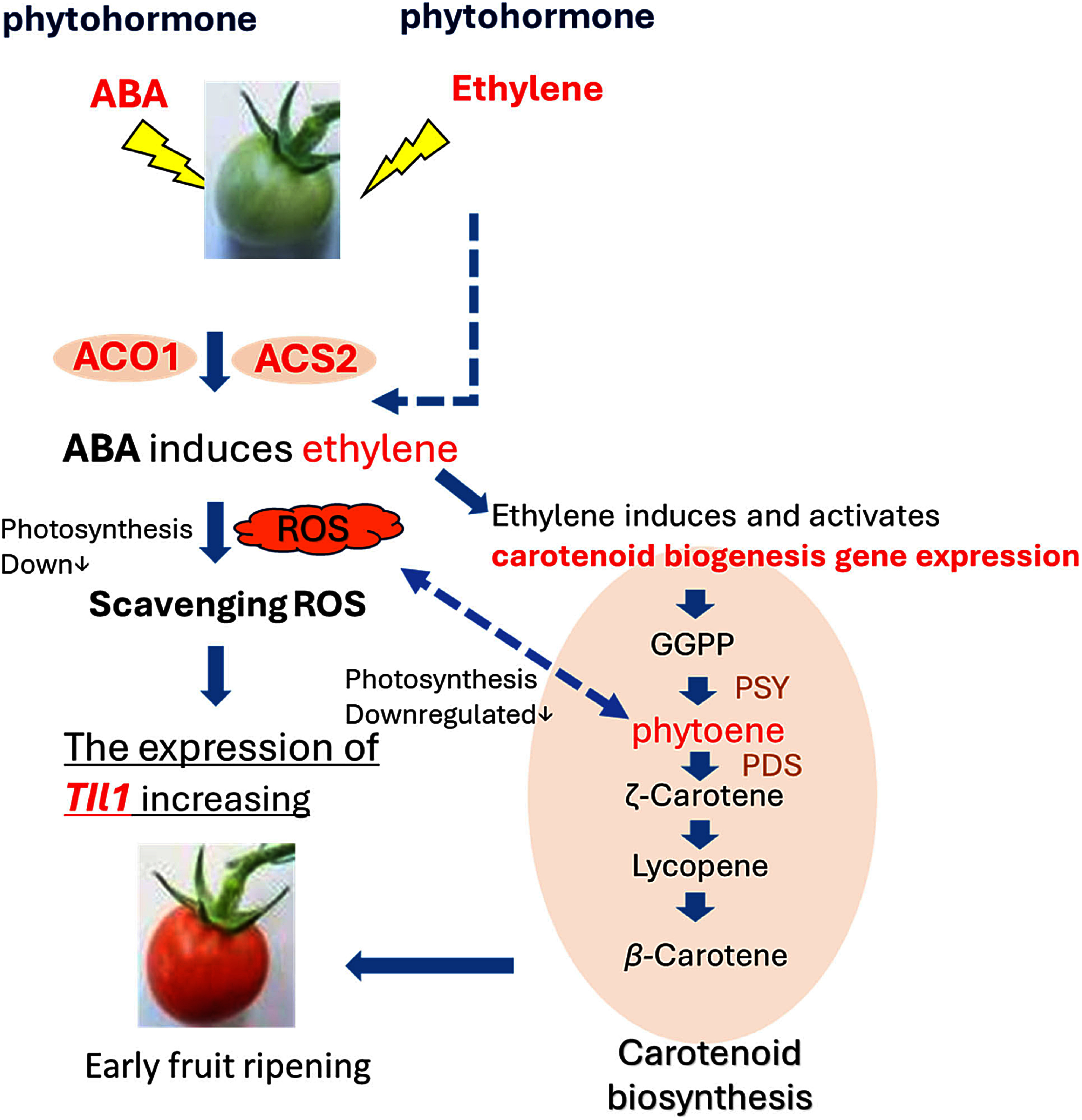
Figure 9. Proposed model of the response of tomato lipocalins to ethylene and abscisic acid (ABA) during fruit ripening. ABA induces ethylene production, which in turn activates the expression of the carotenoid biosynthesis gene *SlPSY1*. This leads to increased phytoene accumulation, followed by a burst of reactive oxygen species (ROS). Lipocalins act positively to scavenge these ROS, thereby enhancing *SlTIL1* expression and promoting early fruit ripening in tomato.

Meanwhile, as carotenoid synthesis is promoted via ethylene biosynthesis, photosynthetic activity is reduced. During this process, *SlPSY1* expression increases, and phytoene gradually accumulates (Llorente et al. 2020), leading to elevated ROS levels. In response, *SlTIL1* is expected to be highly expressed to scavenge the increased ROS (Figure 9). Interestingly, a recent study revealed that phytoene overproduction initially interferes with photosynthesis, acting as a metabolic threshold switch that weakens chloroplast identity. In a subsequent stage, the conversion of phytoene into downstream carotenoids is required for chromoplast differentiation—a process involving the reprogramming of nuclear gene expression and plastid morphology to enhance carotenoid storage (Llorente et al. 2020). Furthermore, ROS production has been shown to stimulate chromoplastogenesis. A previous study also suggested that carotenoid accumulation and ROS generation are not merely consequences but active promoters of chromoplast differentiation (Morelli et al. 2023). These findings indicate that phytoene accumulation and ROS production contribute to chromoplast differentiation, leading to leaf senescence and the early stages of fruit ripening. Therefore, we propose that *SlTILs* play a critical role in scavenging ROS, thereby contributing to the induction of ethylene and ABA responses and the early onset of fruit ripening. Additionally, the binding of the carotenoid astaxanthin in the multimacromolecular protein complex crustacyanin is responsible for the blue coloration of lobster shells. Crustacyanin belongs to the lipocalin superfamily, proteins known to transport steroids, carotenoids, retinoids, and other small hydrophobic molecules (Cianci et al. 2002; Flower 1996; Yao et al. 2023). Yao et al. (2023) further demonstrated that the lipocalin MrLC in crustaceans such as lobsters and prawns possess astaxanthin-binding capacity. Therefore, further elucidation of the TFs regulating lipocalin gene expression, as well as the potential for carotenoid binding by tomato lipocalins, will offer valuable insights into lipocalin function and its role in tomato fruit ripening.

## Description of supplementary files

Supplementary Table S1. Sequence details of the primers used in this study.

Supplementary Figure S1. *Cis*-acting elements are present in the *5*′ upstream genomic regions (1,800 bp) of *SlTIL1*, *SlTIL2*, and *SlCHL*, including the promoter regions.

Supplementary Figure S2. Response of lipocalins to MeJA treatment in leaves.

Supplementary Figure S3. Expression of *SlTIL1* and *SlTIL2* genes following treatment with 100 µM ABA in wild-type (WT) young leaves.

Supplementary Figure S4. Thermal images of wild-type (WT) young leaves under control conditions (no ABA treatment).

Supplementary Figure S5. Number of days to fruit ripening after flowering for fruits treated with 50 μM or 100 μM ABA, compared with wild-type (WT) fruits at the mature green (MG) stage.

Supplementary Figure S6. Expression of ethylene biosynthesis genes in overexpression lines (*SlTIL1*OX, *SlTIL2*OX, and *SlCHL*OX) compared with WT plants.

Supplementary Figure S7. Expression of carotenoid biosynthesis genes in *SlTIL1OX*, and *SlCHLOX* lines compared with WT plants.

Supplementary Figure S8. Structural comparisons of lipocalins (SlTILs and SlCHL) using TMHMM, an in silico tool for predicting transmembrane domains.
