# Supplementary material for: Tomato lipocalins mediate ABA (abscisic acid)- and ethylene-dependent regulation of stress tolerance and fruit ripening
Source: Plant Biotechnol (Tokyo). 2026 Mar 25;43(1):61–71. doi: 10.5511/plantbiotechnology.25.1023b (PMC13170811; doi:10.5511/plantbiotechnology.25.1023b)
Supplement: Supplementary Data [file plantbiotechnology-43-1-25.1023b-s001.pdf]

Supplementary Information

Supplementary Table S1. Primer sequences used in this study.

(1) For expression analysis of lipocalin genes by RT-PCR

**Primer name**	**Primer Sequence (5′ – 3′)**	**Gene Locus**
SlTIL1 Forward	GGCTACAAAAGTAATGGAAGTG	Solyc12g010320
SlTIL1 Reverse	CTTGTGGAGCTTACTCACGTC	
SlTIL2 Forward	ATGACCACAAAAGAGATGGAAGTA	Solyc07g005210
SlTIL2 Reverse	CTATTTTCCCAATATTGATTTGATCC	
SlCHL Forward	CTGTGAAAGTGGTGTCTTCC	Solyc12g005180
SlCHL Reverse	CTTCTTGACCTTGTCCAGCA	
SlActin Forward	GAGCACCCAGTTCTCCTCAC	Solyc10g080500
SlActin Reverse	TAACAATTTCCCGCTCGGCT	

(2) For expression analysis of ethylene biosynthesis genes

**Primer name**	**Primer Sequence (5′ – 3′)**	**Gene Locus**
SlACO1 Forward	GTGAGTGGCCTTCAACTCCT	Solyc07g049530
SlACO1 Reverse	AATTGGATCACTTTCCATTGCC	
SlACS1A Forward	GCCTTTTTGGTCCCAACAACC	Solyc01g095080

SlACS1A Reverse	CCTGCCTTTCTCCGAGTCTT	
SlACS2 Forward	GTGGTGCCACTGGAGCTAAT	Solyc01g095080
SlACS2 Reverse	ACTGACGAATTAAGGCGTGT	
SlACS4 Forward	ATGGGTCTCGCGGAAAATCAG	Solyc05g050010
SlACS4 Reverse	GAATTGAACACGGTAGCAGCA	

(3) For expression analysis of jasmonate ZIM (JAZ)-domain genes.

**Primer name**	**Primer Sequence (5′ – 3′)**	**Gene Locus**
SlJAZ1 Forward	CATGTCCCAACACGCAATGG	Solyc07g042170
SlJAZ1 Reverse	GAAGTACTTGAGCCTGAGG	

(4) For expression analysis of carotenoid biosynthesis genes

**Primer name**	**Primer Sequence (5′ – 3′)**	**Gene Locus**
SlPDS Forward	TAACTGCCAAACCACCACAA	Solyc03g123760
SlPDS Reverse	ACCCATTGATTCGCTACCAG	
SlPSY1 Forward*[1]	TGTATGGGCATCTTTGGTCTTGTA	Solyc03g031860
SlPSY1 Reverse*[1]	CAGTTTTTGTAGGAGGCACAAGAG	
SlNCED1 Forward	TATGGTTCACGCCGTTCAATTCA	Solyc07g056570
SlNCED1 Reverse	AGACTAAACCGGCGTTTGCAACAC	Solyc07g056570
SlNCED2 Forward	AAATCGCCGGAAACTTTGCTC	Solyc08g016720
SlNCED2 Reverse	CGAAAACTCCACGGGCATAGAA	
SlCRTISO Forward	GATCGCCAAATCCTTAGCAA	Solyc10g081650
SlCRTISO Reverse	GCCCTGGGAAGAGTGTTTTT	
SlCycb-2 Forward	CCCTTCAC CACTCTCCATGT	Solyc06g074240
SlCycb-2 Reverse	TCCCTCCAATCCATAAGCAC	

SlVDE Forward	TGTCAAGTTGGATCAAGGCT	Solyc04g050930
SlVDE Reverse	CATCTCAAACCCTGCATGTG	
SlZDS Forward	CAGAAGTGGAGGGAATTGGA	Solyc01g097810
SlZDS Reverse	AGGCATGTAAGGGTCACCAG	
SlZEP Forward	CCTTGTAGGAGCTTGGAAAATGGG	Solyc02g090890
SlZEP Reverse	TCTTATGCCATCAGCACCAACCAG	

*1 *PSY1* is same primer set to real time PCR

(5) For quantitative real-time PCR (qRT-PCR)

**Primer name**	**Primer Sequence (5′ – 3′)**	**Gene Locus**
SlTIL1 Forward	TAAAGATGGAGTGGACACAAGGG	Solyc12g010320
SlTIL1 Reverse	CCTCCTACTAGGCTGACCAATC	
SlTIL2 Forward	ATGGAGTGGTGGTAAAAGAGGTTC	Solyc07g005210
SlTIL2 Reverse	CTTCATCAAGATGTGGTTGCCTAC	
SlPSY1 Forward	TGTATGGGCATCTTTGGTCTTGTA	Solyc03g031860
SlPSY1 Reverse	CAGTTTTTGTAGGAGGCACAAGAG	
SlPDS Forward	CCAAGACCAGAGCTGGACAA	Solyc03g123760
SlPDS Reverse	GCCTCCAGCAGTATCGGTTT	
Sl18SrRNA Forward	GCGCGCTACACTGATGTATTC	NCBI Accession No. OK073663.1
Sl18SrRNA Reverse	GCGATCCGAACATTTCACCG	

SlActin Froward	AATTGGATCACTTTCCATTGCC	Solyc10g080500
SlActin Reverse	ACGCCCTGCAAGATCAAGAC	

Reverse Transcription PCR (RT-PCR) was performed for gene expression analysis, as described in our previous study (Wahyudi et al. 2020).

Wahyudi A, Fukasawa C, Motohashi R (2020) Function of *SlTILs* and *SlCHL* under heat and oxidative stresses in tomato. *Plant Biotechnology* 37(3): 335-341

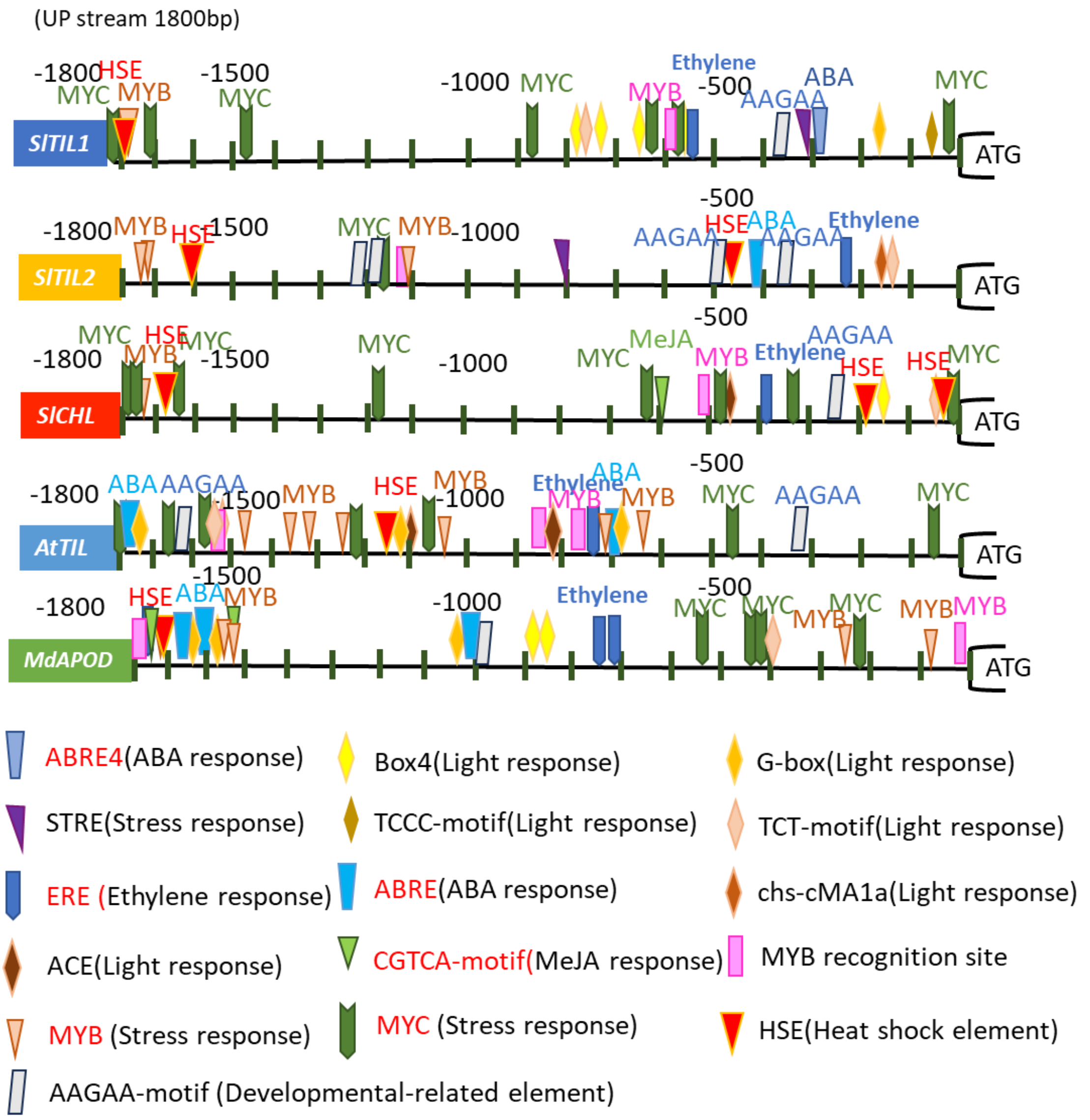

Supplementary Figure S1

Cis-acting elements are present in the *5'* upstream genomic regions (1,800 bp) of *SlTIL1*, *SlTIL2,* and *SlCHL,* including the promoter regions. ABRE4: ABA response; ABRE: ABA response; ERE: ethylene response; MYB: stress response; MYC: stress response; G-box: light response.

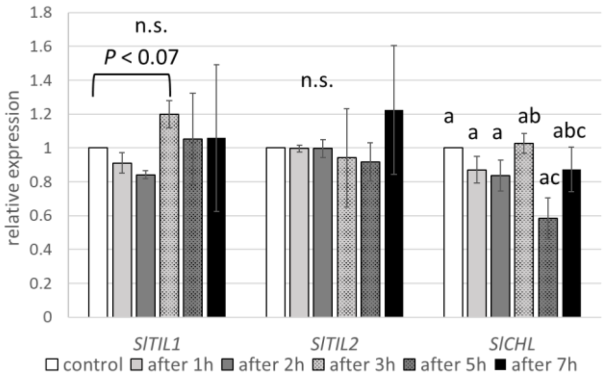

Supplementary Figure S2

Response of lipocalins to MeJA treatment in leaves. Expression levels of *SlTILs* and *SlCHL* were analyzed following treatment with 38.6 μM MeJA in wild-type (WT) young leaves. Expression was measured by RT-PCR (see Supplementary Table S1). Each value represents the mean ± SD of three biological replicates. Different letters indicate statistically significant differences ($p < 0.05$) based on Tukey's multiple comparisons test; n.s., not significant ($p > 0.05$).

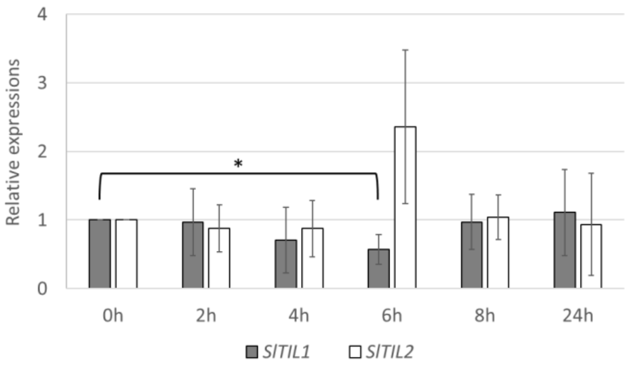

Supplementary Figure S3

Expression of *SlTIL1* and *SlTIL2* genes following treatment with 100 µM ABA in wild-type (WT) young leaves. Expression levels were measured by RT-PCR. Each value represents the mean ± SD of three biological replicates. Asterisk represent statistically significant differences compared with 0 h, as determined by a two-tailed Student's *t*-test (*$p < 0.05$).

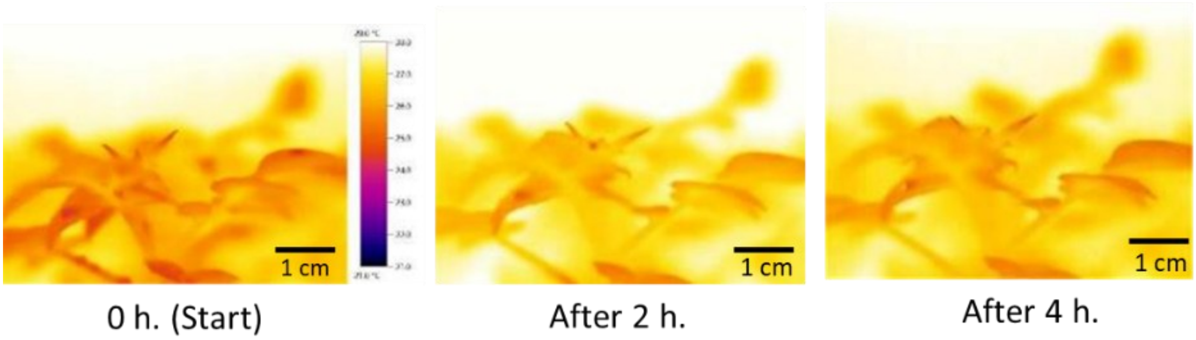

Supplementary Figure S4

Thermal images of wild-type (WT) young leaves under control conditions (no ABA treatment). The entire leaf surface was sprayed with distilled water from a height of 10 cm for full coverage. The scale bar represents 1 cm.

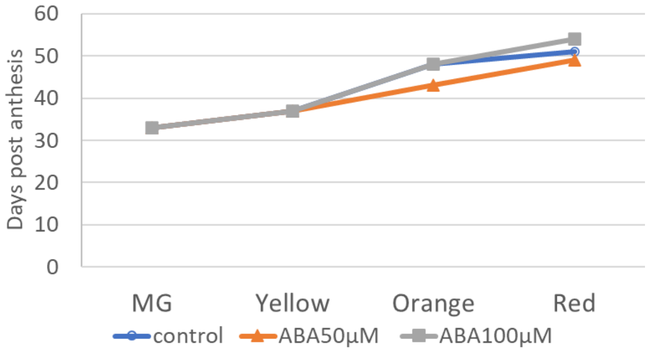

Supplementary Figure S5

Number of days to fruit ripening after flowering for fruits treated with 50 µM or 100 µM ABA, compared with wild-type (WT) fruits at the mature green (MG) stage.

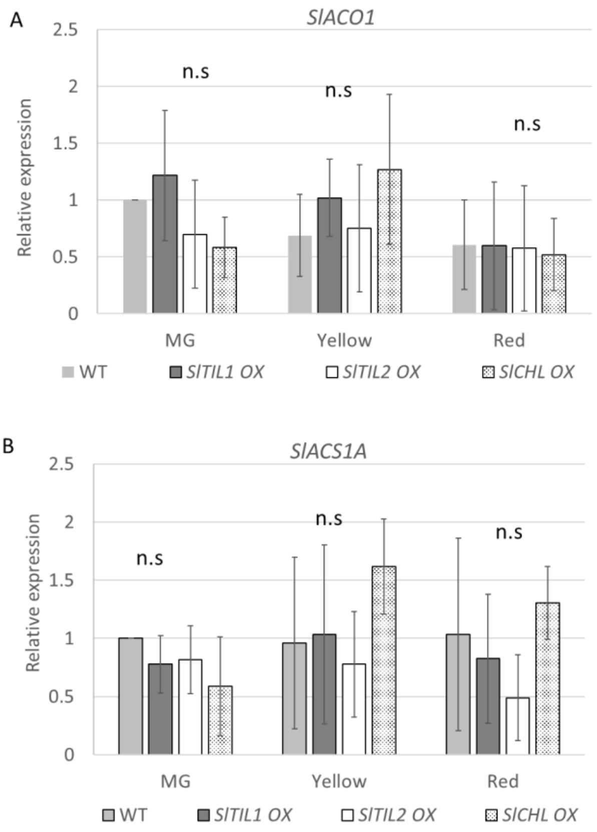

Supplementary Figure S6

C

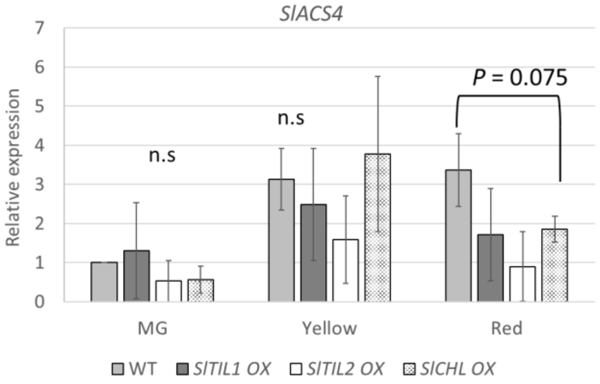

Supplementary Figure S6

Supplementary Figure S6.

Expression of ethylene biosynthesis genes in overexpression lines (*SlTIL1*OX, *SlTIL2*OX, and *SlCHL*OX) compared with WT plants. (A) *SlACO1*, (B) *SlACS1A*, (C) *SlACS4*. Expression levels were analyzed by RT-PCR. Each value represents the mean ± SD of three biological replicates. Different letters indicate statistically significant differences ($p < 0.05$) based on Tukey's multiple comparisons test; n.s. means not significant ($p > 0.05$).

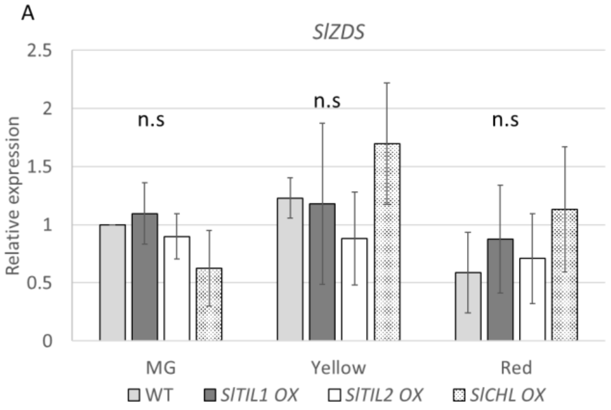

Supplementary Figure S7

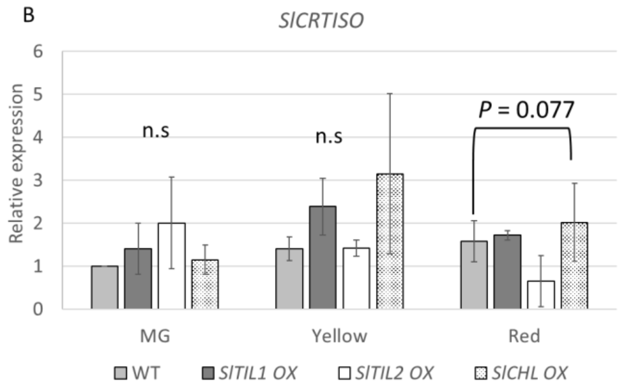

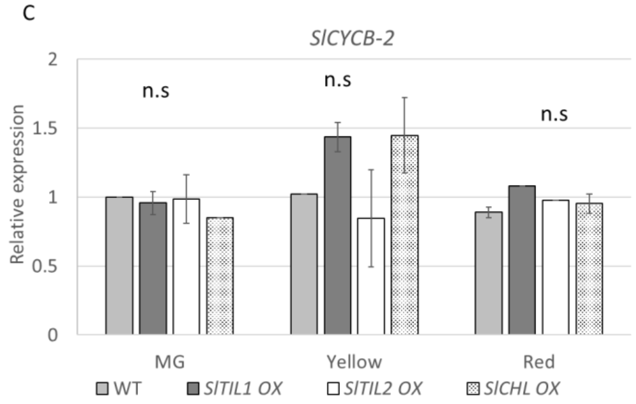

Supplementary Figure S7

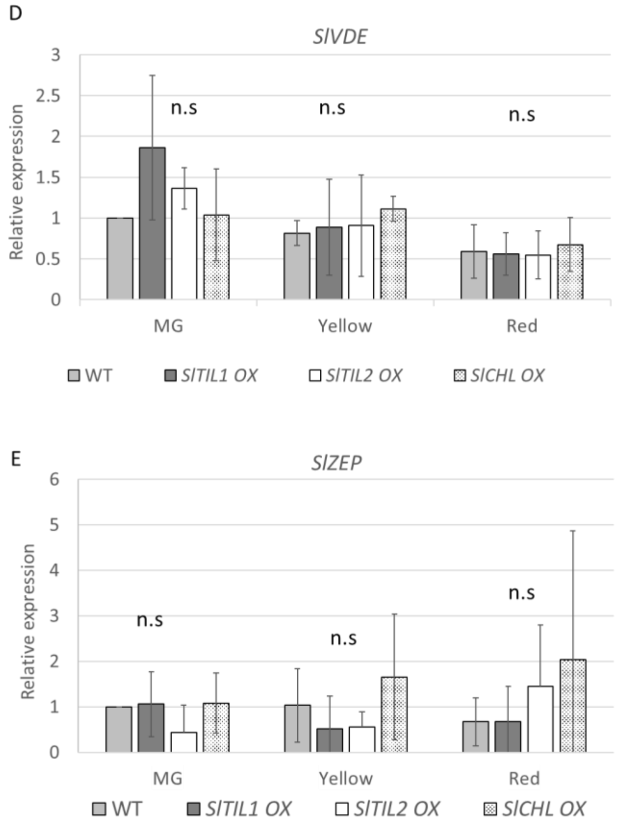

Supplementary Figure S7

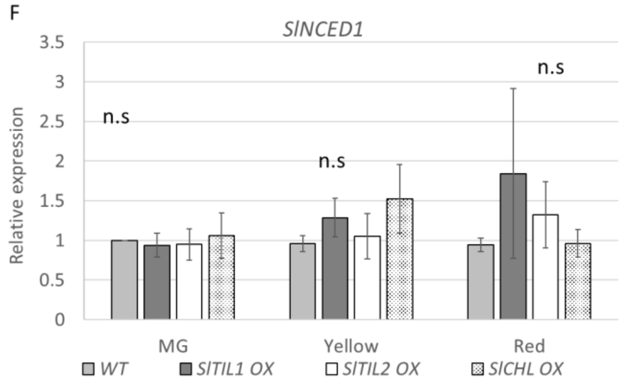

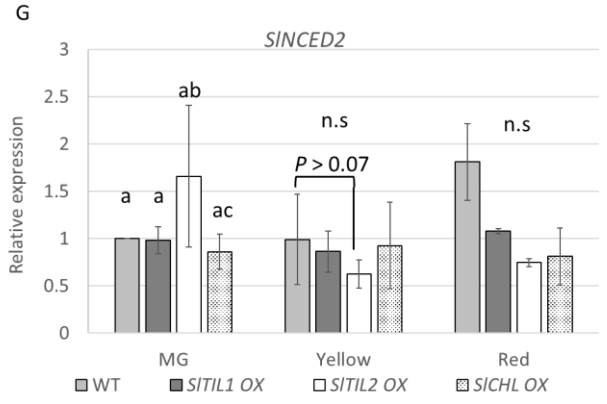

Supplementary Figure S7

Supplementary Figure S7.

Expression of carotenoid biosynthesis genes in *SlTIL1OX*, and *SlCHLOX* lines compared with WT plants. (A) *SlZDS,* (B) *SlCRTISO*, (C) *SlCYCB-2*, (D) *SlVDE*, (E) *SlZEP,* (F) *SlNCED1, and* (G) *SlNCED2*, and . Expression levels were analyzed by RT-PCR. Each value represents the mean ± SD of three biological replicates. For panels A, B, D, E, F, and G, different letters indicate statistically significant differences ($p < 0.05$) based on Tukey's multiple comparisons test; n.s., not significant ($p > 0.05$). Data in panel C were analyzed using a two-tailed Student's *t-test*.

A

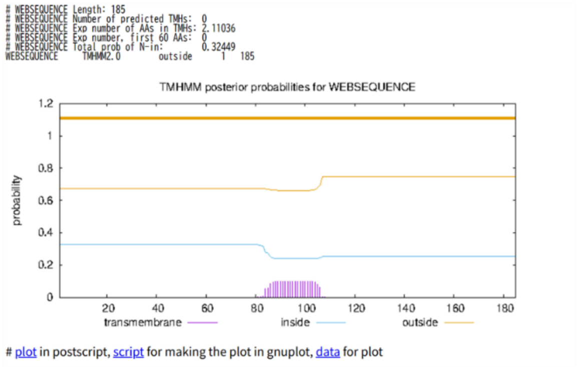

B

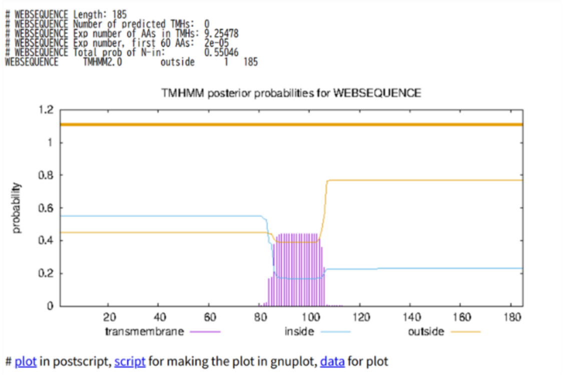

C

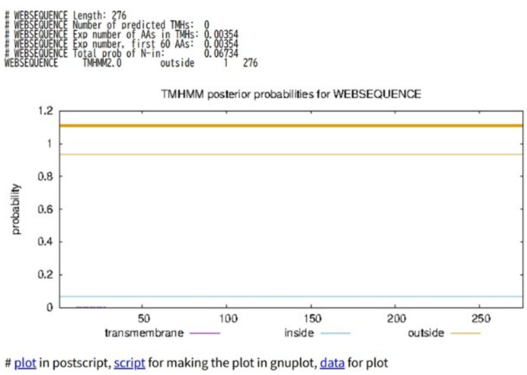

Supplementary Figure S8

Structural comparisons of lipocalins (SlTILs and SlCHL) using TMHMM, an in silico tool for predicting transmembrane domains. (A) SlTIL1, (B) SlTIL2, (C) SLCHL.
